# Tubulopathy meets Sherlock Holmes: biochemical fingerprinting of disorders of altered kidney tubular salt handling

**DOI:** 10.1007/s00467-021-05098-5

**Published:** 2021-06-18

**Authors:** Detlef Bockenhauer, Robert Kleta

**Affiliations:** 1 Department of Renal Medicine, University College London, NW3 2PF, London, UK; 2 Great Ormond Street Hospital for Children NHS Foundation Trust, London, UK

**Keywords:** Salt-wasting tubulopathies, Salt-retaining tubulopathies, Distal renal tubular acidosis, Renal Fanconi Syndrome, Bartter Syndromes, Gitelman Syndrome, EAST Syndrome, Pseudohypoaldosteronism, Liddle syndrome, Apparent Mineralocorticoid Excess

## Abstract

Evolution moves in mysterious ways. Excretion of waste products by glomerular filtration made perfect sense when life evolved in the ocean. Yet, the associated loss of water and solutes became a problem when life moved onto land: a serious design change was needed and this occurred in the form of ever more powerful tubules that attached to the glomerulus. By reabsorbing typically more than 99% of the glomerular filtrate, the tubules not only minimise urinary losses, but, crucially, also maintain homeostasis: tubular reabsorption and secretion are adjusted so as to maintain an overall balance, in which urine volume and composition matches intake and environmental stressors. A whole orchestra of highly specialised tubular transport proteins is involved in this process and dysfunction of one or more of these results in the so-called kidney tubulopathies, characterised by specific patterns of clinical and biochemical abnormalities. In turn, recognition of these patterns helps establish a specific diagnosis and pinpoints the defective transport pathway. In this review, we will discuss these clinical and biochemical “fingerprints” of tubular disorders of salt-handling and how sodium handling affects volume homeostasis but also handling of other solutes.

## Introduction

The function of our body is critically dependent on a stable “internal milieu”. The term was coined by the French physiologist Claude Bernard about 150 years ago, who recognised the critical importance of this internal milieu for “a free and independent existence” and that therefore “all the vital mechanisms, however varied they may be, have only one object, that of preserving constant the conditions of life in the internal environment” [1]. Subsequently, the American physiologist Walter Cannon called this process of maintaining the internal milieu “homeostasis” [2]. The recognition of the importance of homeostasis and how it evolved was further detailed by Homer Smith in his book *From fish to philosopher*—without the tightly regulated concentrations of electrolytes that allows muscles to contract, nerves to fire, hormones to be secreted and enzymes to catalyse, we would not be able to function, let alone become philosophers [3]. Human kidneys initially produce a large volume of primary urine by glomerular filtration, of which about 99% needs to be reabsorbed back in to the blood by tubular transport. This process of first “leaking” and then reabsorbing large amounts of salt and water may not appear being “intelligent design”, but is explained by the evolutionary beginning in the ocean, where saltwater availability was unlimited. With emergence of life onto land, the existing process was progressively adapted to conserve salt and water by the evolution of ever more powerful tubules. Specific transporters developed to reabsorb their respective cargo along the tubules and the reabsorption of sodium gained an especially prominent role in kidney tubular function. This is due to the critical importance of sodium in the maintenance of volume homoeostasis. Moreover, the electrochemical gradient for sodium, established by the sodium–potassium ATPase is used as a driving force for many other transport processes, such as sodium co-transport (e.g. glucose, phosphate, amino acids) or sodium exchange (e.g. protons, potassium). Several diseases have been described due to defects in tubular sodium transport. Due to the specific transport characteristics of the affected tubular segments, each of these disorders is associated with a specific biochemical pattern in the plasma and urine electrolytes. This review aims to characterise these patterns and explain how they can be used as diagnostic “fingerprints” to identify the underlying transport defect and thus guide proper diagnosis, which can subsequently be confirmed by genetic testing [4], and appropriate treatment. One key insight from these disorders of renal salt handling is that they rarely affect the sodium concentration in blood, but rather volume homeostasis: salt-wasting disorders are associated with hypovolaemia and lower blood pressure. Conversely, salt-retaining disorders result in hypervolaemia and hypertension [5]. However, the biochemical fingerprints in isolation do not necessarily establish a diagnosis, but must be interpreted in the clinical context, as will become evident in the discussion of the patterns of hypokalaemic alkalosis and hyperkalaemic acidosis, both of which can be associated with salt-wasting, as well as salt-retaining disorders. The distinction between these opposite states is therefore made by clinical examination, i.e. assessment of volume status, rather than biochemical investigations [6]. An overview of the specific patterns and their associated disorders is given in Table 1.

**Table 1:**
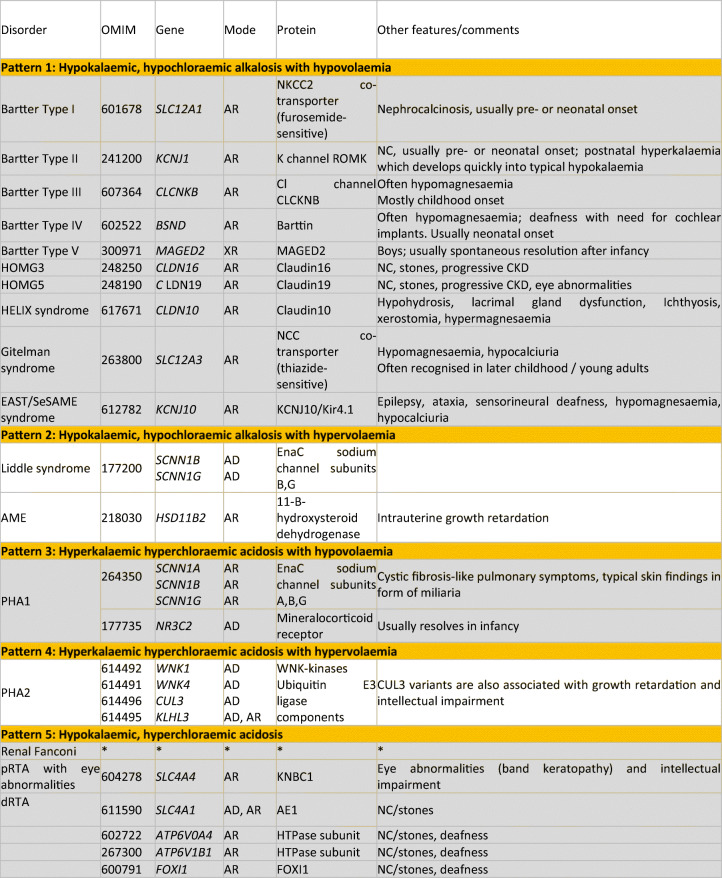
Clinical and molecular characteristics of renal salt-handling disorders

Listed are disorders of tubular salt handling (adapted from [7] with permission). The shaded rows list disorders of salt wasting (hypovolaemia and low-normal BP). Not included are endocrine disorders with secondary effect on salt handling, such as primary hyperaldosteronism and the congenital adrenal hyperplasias

*Renal Fanconi syndrome typically occurs secondary, such as in cystinosis, but there are also primary forms recognised; HOMG3 and HOMG5 are also known as Familial Hypomagnesaemia with Hypercalciuria and Nephrocalcinosis; *NC* Nephrocalcinosis, *d* decreased, *i* increased, *n* normal, *Aldo* aldosterone, *AME* apparent mineralocorticoid excess, *GRA* glucocorticoid-remediable aldosteronism, *AR* autosomal recessive, *AD* autosomal dominant, *XR* X-linked recessive

### Pattern 1: Hypokalaemic, hypochloraemic alkalosis with hypovolaemia

**Fig. 1:**
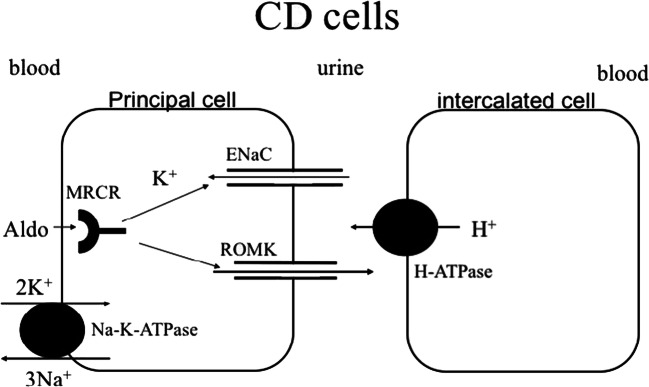
Diagram of a principal and type A intercalated cell in the collecting duct (CD)**. S**odium is reabsorbed in the CD via the epithelial sodium channel ENaC, which is blocked by amiloride and mutated in the recessive form of pseudohypoaldosteronism type 1 (PHA1). Uptake of sodium creates a favourable gradient for potassium secretion and proton secretion (from the adjacent intercalated cells). Expression of ENaC in the membrane is controlled by the mineralocorticoid receptor (MRCR), mutated in the dominant form of PHA1. In the functional collecting duct, stimulation by aldosterone thus leads to hypokalaemic alkalosis. Adapted from [8], with permission

Hypokalaemic, hypochloraemic alkalosis is probably the best recognised biochemical pattern, as it is associated with arguably the most common kidney tubulopathies, Bartter and Gitelman syndromes. Notably, while Bartter’s and Gitelman syndromes are due to impaired salt reabsorption in the thick ascending limb of Henle (TAL) and distal convoluted tubule (DCT), respectively, the characteristic biochemical fingerprint is actually generated downstream in the collecting duct (CD): in a desperate attempt to reclaim as much as possible of the sodium not reabsorbed upstream, the epithelial sodium channel ENaC is upregulated in the apical membrane of the principal cells of the collecting duct, through which sodium enters the cell following the electrochemical gradient established by the basolateral Na-K-ATPase. The electrical balance is maintained by secretion of potassium via KCNJ1 (also called ROMK) and protons via the H-ATPase in the neighbouring type A intercalated cell into the tubular lumen, thereby generating the hypokalaemic metabolic alkalosis (Figure 1). This transport pathway is under the influence of aldosterone and therefore disorders associated with increased aldosterone levels are typically characterised by this biochemical fingerprint. Note that sodium transport in TAL (via the co-transporter NKCC2/SLC12A1) or DCT (via NCC/SLC12A3) is in the form of sodium chloride, whereas in the CD it is reabsorbed in isolation with consequent increased excretion of potassium and hydrogen chloride, contributing to the hypochloraemia. Indeed, it is the increased excretion of chloride that characterises the salt-wasting disorders of Bartter and Gitelman syndromes [9].

### Pattern 1a: Hypokalaemic, hypochloraemic alkalosis with hypovolaemia and hypomagnesaemia

The additional presence of hypomagnesaemia can help to distinguish between salt wasting in TAL (usually normomagnesaemia) or DCT (usually hypomagnesaemia), highlighting the critical role of DCT for magnesium homeostasis [10]. How exactly sodium chloride and magnesium transport are linked in DCT is still unclear, but presumably this is through the activity of the basolateral Na-K-ATPase. This “engine” of tubular transport generates a high intracellular potassium concentration, which in turn helps establish a lumen-positive potential via an apical potassium conductance, which in turn facilitates magnesium uptake via the magnesium channel TRPM6. Conditions that impair the activity of the Na-K-ATPase, either because of decreased supply of sodium (Gitelman syndrome) or potassium (EAST syndrome) or altered regulation (pathogenic variants in *FXYD2* or *HNF1B*) or direct loss-of-function (pathogenic variants in ATP1A1) or impaired energy supply (mitochondrial diseases) can therefore also affect magnesium transport in DCT [10].

There are, however, exceptions to this: Familial hypomagnesaemia with hypercalciuria and nephrocalcinosis (FHHNC) is a salt-wasting disorder of the TAL, due to impaired paracellular transport of cations in this segment [11]. It is associated with hypokalaemic, hypochloraemic alkalosis, albeit usually mild and this pattern may be lost with advanced chronic kidney disease, but also hypomagnesaemia. This disorder thus highlights the importance of the TAL for magnesium reabsorption, where, in fact, the majority of filtered magnesium is reabsorbed [12]. Yet, why there is pronounced hypomagnesaemia in this TAL disorder, but not, for instance in Bartter syndrome types 1 and 2, which also impair paracellular transport, remains a mystery. Thus, our tubular Sherlock Holmes will have to make use of additional clues: hypercalciuria and nephrocalcinosis. The presence of these features strongly suggests a disorder of TAL, irrespective of plasma magnesium levels, whereas hypocalciuria is consistent with a disorder of DCT [13].

Lastly, it is important to note that hypomagnesaemia in salt-wasting disorders of the DCT develops during childhood and typically becomes apparent in the second decade of life [14, 15]. The absence of hypomagnesaemia in a young child therefore does not exclude these disorders.

### Pattern 2: Hypokalaemic, hypochloraemic alkalosis with hypervolaemia

Importantly, the biochemical pattern hypokalaemic, hypochloraemic alkalosis alone does not provide a diagnosis, but must be seen in the context of clinical signs. As detailed above, it is associated with increased aldosterone, but this can be primary (as in Conn syndrome) or secondary (as in Bartter and Gitelman syndromes, but also in renal artery stenosis). Therefore, hypokalaemic, hypochloraemic alkalosis can be due to increased aldosterone in both salt-wasting as well as salt-retaining disorders and biochemical clues alone are not sufficient to distinguish between those. Rather, clinical examination is needed to assess volume status. Moreover, this biochemical pattern can also be seen with suppressed aldosterone in disorders mimicking aldosterone activity, including Liddle syndrome (due to gain-of-function variants in ENaC) or activation of the mineralocorticoid receptor by other steroid hormones, such as progesterone in pregnancy-associated hypertension (due to a variant that enhances sensitivity of the mineralocorticoid receptor to progesterone) or cortisol as in Apparent Mineralocorticoid Excess (due to loss of protection of the mineralocorticoid receptor from cortisol by the enzyme HSD11B2) [16].

Of note, these salt-retaining disorders are typically associated with hypercalciuria. Yet, in contrast to the salt-wasting disorders Bartter syndrome and FHHNC, where hypercalciuria is due to impaired calcium reabsorption in TAL, hypercalciuria in salt-retaining states reflects decreased proximal reabsorption: the hypervolaemia leads to a compensatory decrease in proximal salt reabsorption, which in turn also decreases the concomitant calcium reabsorption. Moreover, the hypercalciuria and/or hypokalaemia is associated with a urinary concentrating defect (secondary nephrogenic diabetes insipidus) and thus polyuria [17]. This again highlights the importance of clinical assessment of volume status to distinguish for instance between a child with Bartter syndrome or Apparent Mineralocorticoid Excess, who can have almost identical biochemical features [18].

### Pattern 3: Hyperkalaemic hyperchloraemic acidosis with hypovolaemia

Hyperkalaemic hyperchloraemic acidosis is the mirror image of the hypokalaemic hypochloraemic alkalosis discussed above and indeed also the aetiology is mirrored, whereas hypokalaemic hypochloraemic alkalosis indicates enhanced ENaC-mediated sodium reabsorption in the collecting duct, hyperkalaemic hyperchloraemic acidosis reflects decreased sodium flux through ENaC. Because of electroneutrality, impaired sodium reabsorption in CD will impede potassium and proton secretion, leading to the hyperkalaemic acidosis. If associated with hypovolaemia, there is primary impaired ENaC-mediated sodium reabsorption and this is referred to as Pseudohypoaldosteronism type 1 (PHA1). There are two inherited forms of this: (1) an autosomal dominant form, due to loss-of-function variants in the mineralocorticoid receptor, and (2) an autosomal recessive form due loss-of-function variants in the genes encoding the three subunits of ENaC (Table 1). In addition, there is an acquired form associated with severe pyelonephritis and/or urinary obstruction [19]. Interestingly, PHA1 is the only salt-wasting tubulopathy associated with hyponatraemia, presumably because the hypovolaemia triggers release of anti-diuretic hormone and with urinary concentration intact, the low solute intake of milk-fed babies in conjunction with a concentrated urine leads to progressive hyponatraemia, aggravated by the urinary sodium losses [20].

### Pattern 4: Hyperkalaemic hyperchloraemic acidosis with hypervolaemia

While hyperkalaemic hyperchloraemic acidosis with a primary impairment of ENaC-mediated sodium reabsorption is a salt-wasting condition, there is also a fascinating salt-retaining condition called Pseudohypoaldosteronism type 2 (PHA2 or Gordon syndrome) in which there is enhanced sodium reabsorption in the DCT with consequent decreased delivery to the CD, where the sodium could be exchanged for potassium or protons [21]. Despite the similar biochemical profile, PHA1 and PHA2 are rarely confused. Patients with PHA1 typically present in early infancy with severe, even life-threatening hypovolaemia, whereas PHA2 is usually diagnosed later in childhood or adulthood with hypertension as the main presenting syndrome. A rare autosomal recessive form of PHA2 (due to pathogenic variants in *KLHL3*) may also present early in infancy, but again the increased blood pressure in this condition makes it easily distinguishable from PHA1. In addition, there is a biochemical clue: because of the hypervolaemia, PHA2 is associated with hypercalciuria (due to decreased proximal reabsorption, see *Pattern 2* above), whereas in PHA1 with the associated hypovolaemia there is hypocalciuria.

Identification of the molecular basis of PHA2 shed light on the “aldosterone paradox”, which refers to the dual roles of aldosterone in volume as well as potassium homeostasis. These roles can at times be conflicting, for instance if both, i.e. volume (sodium) and potassium need to be preserved. The genes causative for PHA2 all work in a pathway that shunts sodium reabsorption to the DCT, if both sodium and potassium need to be preserved, and to the CD, if potassium must be excreted [22]. In this way, the DCT also serves as a potassium sensor for overall regulation of potassium homeostasis [23].

### Pattern 5: Hypokalaemic, hyperchloraemic acidosis

The pattern of hypokalaemic, hyperchloraemic acidosis is the fingerprint of renal tubular acidosis (RTA), both proximal (pRTA, also called RTA “type 2”) and distal (dRTA or RTA “type 1”). While these are primarily disorders of acid-base homeostasis, sodium reabsorption is affected as well, and therefore, these disorders are included here.

In pRTA, the acidosis is due the impaired reabsorption of sodium-bicarbonate in the proximal tubule. Typically, this is in the context of a generalised proximal tubular dysfunction (renal Fanconi syndrome) [24]. Isolated pRTA is exceedingly rare and associated with eye abnormalities, typically band keratopathy [25]. This disorder is due to pathogenic variants in the basolateral sodium bicarbonate co-transporter SLC4A4 [26]. The aetiology of the variable hypokalaemia in renal Fanconi syndrome is probably two-fold: (1) impaired proximal potassium reabsorption and (2) activation of the renin-aldosterone system because of salt and volume losses with consequent enhanced distal potassium secretion. While the activation of the renin-aldosterone system will also enhance distal proton secretion (see patterns 1 and 2 above), this does not result in alkalosis here, as the excess protons secreted distally are negligible compared to the amount of bicarbonate not reabsorbed proximally.

While acidosis in pRTA is caused by urinary bicarbonate loss, in dRTA acidosis stems from the failure to secrete protons with consequent bicarbonate loss from buffering the retained protons [13]. And while the fingerprint of hypokalaemic, hyperchloraemic acidosis is identical in both forms, there are sufficient other clues for the tubular Sherlock Holmes to readily distinguish between them, e.g. urine pH—once plasma bicarbonate levels have fallen below the threshold for proximal tubular reabsorption, a new steady state is reached, bicarbonate wasting stops and with distal urine acidification intact, urine pH can reach levels below 5.3 [27]. In contrast, in dRTA urine pH is always > 5.3, in fact, typically > 7. Moreover, since pRTA typically occurs in the context of renal Fanconi syndrome, there are other biochemical abnormalities, such as hypophosphataemia with renal phosphate wasting, as well as glycosuria, low molecular weight proteinuria and aminoaciduria that help identify the correct diagnosis. And even in the rare cases of isolated pRTA, the diagnosis can be easily reached by typical associated eye findings. Urinary calcium excretion, however, can only distinguish between proximal and distal RTA once treatment has been started. In dRTA hypercalciuria is due to acid-mediated calcium reabsorption from the bone, whereas in pRTA there is also impaired proximal tubular calcium reabsorption as part of the renal Fanconi syndrome. Thus, hypercalciuria characterises both forms of RTA. However, once treatment has started and acidosis resolves, calcium excretion in dRTA normalises, whereas it remains elevated in renal Fanconi syndrome.

## Conclusions

Disorders of renal tubular sodium handling can be easily identified by typical biochemical and clinical “fingerprints”. Broad categories are established by the plasma electrolyte profiles and the diagnosis can be further refined by clinical assessment of volume status and by urine biochemistries, such as urinary calcium excretion. Recognising and understanding the aetiology of these patterns not only supports the correct diagnosis, but also helps understand the pathophysiology of these disorders, which, in turn, informs treatment. While genetic testing has come a long way in diagnosing kidney tubulopathies, a genetic diagnosis is established in about 60–80% of patients, mainly because of uncertainties in variant interpretation [28, 29]. Certainty about the clinical diagnosis is often critical for establishing a correct genetic diagnosis and the increasing availability of genetic testing does not relieve us from doing our clinical “due diligence” [30].

### Key summary points

- Tubular disorders of renal salt handling primarily affect volume homeostasis: renal salt-wasting disorders are associated with hypovolaemia and salt-retaining disorders with hypervolaemia.
- Plasma sodium concentration is typically normal in disorders of renal tubular sodium handling.
- Tubular disorders of renal salt handling have characteristic biochemical and clinical features that facilitate diagnosis and understanding of its pathophysiology.
- While clinical features and plasma biochemistries are sufficient to identify the general diagnosis, recognition of specific subtypes also requires urine biochemistries.
- Correlation of clinical features/diagnosis with genetic findings is critical for establishment of the correct diagnosis.

### Multiple Choice Questions (answers given following the reference list)

#### Question 1

Below is a table of biochemistries found in a 2-year-old boy investigated for growth failure. Height and weight are at the 0.4 percentile and blood pressure is 78/46 mmHg.

**Table:** 

Biochemistries	Plasma	Urine	Unit	Remarks
Sodium	137	25	mmol/l	FENa: <1%
Potassium	3.1	17	mmol/l	FEK: 21%
Chloride	113	32	mmol/l	FECl: 1 %
Bicarbonate	16		mmol/l	Urine pH: 5.5
Calcium	2.45	2.3	mmol/l	UCa/UCr: 1.9 mmol/mmol
Phosphate	1.02	17.5	mmol/l	TRP: 35%
Albumin	39	0.153	g/l	UA/UCr: 127 mg/mmol
Urea	5		mmol/l	
Creatinine	0.045	1.2	mmol/l	
Retinol binding protein (RBP)		48.000	mcg/l	RBP/UCr: 40.000 mcg/mmol

- Which of the following statements is correct?
  - The pattern of hypokalaemic hypochloraemic acidosis is characteristic for a defect in the distal convoluted tubule
  - The low-normal blood pressure indicates a salt-retaining syndrome
  - The low plasma phosphate and the low-molecular weight proteinuria (urinary RBP) indicate a defect in the proximal tubule
  - The albuminuria indicates a glomerular defect

#### Question 2

A 2-week-old ex 34-week premature baby is referred because of persistent electrolyte abnormalities. The pregnancy was complicated by polyhydramnios and the mother underwent a total of 3 amniotic fluid aspirations. During the third one, she went into premature labour. The patient’s birth weight was 1.75 kg and she initially required intensive care with fluid administration of up to 300 ml/kg/d. On examination, her weight is 1.76 kg, length: 47 cm, BP: 48/palp mmHg. Biochemistries are detailed below.

**Table:** 

Biochemistries	Plasma	Urine	Unit	Remarks
Sodium	135	30	mmol/l	FENa: 1%
Potassium	3.1	60	mmol/l	FEK: 103% TTKG: 17
Chloride	90	60	mmol/l	FECl: 3.6%
Bicarbonate	28		mmol/l	
Urea	17.9		mmol/l	
Creatinine	0.096	1.8	mmol/l	
Calcium	2.75	4.1	mmol/l	UCa/UCr: 2.3

- Which of the following statements is wrong:
  - The pattern of hypokalaemic hypochloraemic alkalosis is characteristic for a defect in salt reabsorption in TAL or DCT
  - The low-normal blood pressure indicates a salt-wasting syndrome
  - The elevated calcium-creatinine ratio is characteristic for Bartter syndrome types 1 and 2
  - The normal fractional excretion of sodium (FENa) excludes a salt-wasting disorder

#### Question 3

A 6-day-old girl presents acutely with irritability, vomiting and lethargy. Pregnancy was uncomplicated and she was born at term with a birth weight of 2.66 kg. Parents are first cousins and there is a history of early child loss in the extended family.

Examination: weight 2.35 kg, BP: 56/palp, poor skin turgor.

Urine dip: neg. for protein, blood and glucose; pH 8.0, SG: 1.020.

Biochemistries are detailed below:

**Table:** 

Biochemistries	Plasma	Urine	Unit	Remarks
Sodium	132	90	mmol/l	FENa: 5%
Potassium	10.7	8	mmol/l	FEK: 5% TTKG: <1
Chloride	110		mmol/l	
Bicarbonate	12		mmol/l	
Urea	17		mmol/l	
Creatinine	0.086	1.2	mmol/l	
Renin	280		pmol/l/h	(normal <25)
Aldosterone	92000		pmol/l	(normal <2000)

- Which of the following statements are correct? (select all that apply)
  - The fractional excretion of potassium and the transtubular potassium gradient indicate an appropriate tubular response to hyperkalaemia and the elevated aldosterone
  - The clinical hypovolaemia and the hyperkalaemic hyperchloraemic acidosis with dramatically elevated renin and aldosterone are typical for Pseudohypoaldosteronism type 1
  - The constellation of clinical and biochemical findings can be seen in the context of pyelonephritis and/or urinary obstruction
  - Uraemia is an intrinsic part of the disorder
  - Affected children may also suffer from pulmonary and skin manifestations

####  Question 4

A 3-month-old boy presents with growth failure. Height and weight are at the 0.4^th^ percentile. He has several wet nappies per day. On examination, he has dry mucous membranes and a blood pressure of 68/42 mmHg. His urine dip is unremarkable with a pH of 8.0 and a specific gravity of 1.005. His biochemistries are below:

**Table:** 

Biochemistries	Plasma	Urine	Unit	Remarks
Sodium	148	15	mmol/l	FENa: 0.5%
Potassium	2.7	34	mmol/l	FEK: 60%
Chloride	128		mmol/l	
Bicarbonate	12		mmol/l	
Calcium	2.48	2.3	mmol/l	UCa/UCr: 2.9
Urea	5		mmol/l	
Creatinine	0.038	0.8	mmol/l

- Which of the following statements is correct?
  - The biochemical pattern of hypokalaemic, hyperchloraemic acidosis is typical for a defect in DCT
  - The elevated plasma sodium concentration excludes a salt-wasting disorder
  - This disorder can be associated with sensorineural deafness
  - Nephrocalcinosis is rarely associated

#### Question 5

A 15-month-old girl presents with growth failure. Her past medical history is remarkable for intrauterine growth retardation and prematurity (35 weeks) with a birth weight of 1.7 kg. She has frequent wet nappies. On examination, height and weight are at below the 0.4^th^ percentile and blood pressure is 122/76 mmHg. Her urine dip is unremarkable with a pH of 7.0 and a specific gravity of 1.000. Her biochemistries are below:

**Table:** 

Biochemistries	Plasma	Urine	Unit	Remarks
Sodium	146	20	mmol/l	FENa: 0.8%
Potassium	2.6	32	mmol/l	FEK: 74%
Chloride	96	50	mmol/l	FECl: 0.3%
Bicarbonate	29		mmol/l	
Urea	2.9		mmol/l	
Creatinine	0.036	0.6	mmol/l	
Calcium	2.45	2.1	mmol/l	UCa/UCr: 3.5

- Which of the following statements is correct?
  - The history of prematurity and polyuria and the biochemical pattern of hypokalaemic, hypochloraemic alkalosis and the hypercalciuria establish a diagnosis of Bartter’s syndrome in this girl
  - The biochemical pattern of hypokalaemic, hypochloraemic alkalosis indicates a problem in the proximal tubule
  - The normal fractional excretion of sodium (FENa) excludes a salt-retaining disorder
  - The elevated blood pressure suggests a salt-retaining disorder

#### Answers:

-
  1. The low plasma phosphate and the low molecular weight proteinuria (urinary RBP) indicate a defect in the proximal tubule, as both solutes are exclusively reabsorbed in the proximal tubule. This patient has renal Fanconi syndrome. The pattern of hypokalaemic hypochloraemic acidosis is typical for renal tubular acidosis, which is a defect either in the proximal tubule (as in this case) or in the collecting duct. The low-normal blood pressure indicates hypovolaemia and thus salt wasting. While albumin is often used as an indicator of glomerular dysfunction, some albumin is filtered physiologically and reabsorbed in the proximal tubule. Renal Fanconi syndrome is therefore associated also with albuminuria, yet usually below the nephrotic range and without oedema.
  2. The normal fractional excretion of sodium (FENa) excludes a salt-wasting disorder. This boy has a typical history, as well as clinical and biochemical findings for Bartter syndrome (types 1 or 2), a disorder of the TAL and associated with hypercalciuria. The elevated plasma urea and creatinine reflect hypovolaemia and consequent acute kidney injury. In a steady state, urinary sodium excretion must reflect sodium intake; for this reason, the FENa is usually normal in salt-wasting disorders; however, chloride excretion is typically elevated (> 0.5%) [7].
  3. Clinical and biochemical features are classic for Pseudohypoaldosteronism type 1 (PHA1), characterised by hyperkalaemic hyperchloraemic acidosis with impaired urinary potassium and proton excretion, indicating a defect in salt reabsorption in the collecting duct. Uraemia is not an intrinsic part of the syndrome, but reflects AKI from hypovolaemia, and the elevated FENa indicates ongoing volume contraction and thus a high risk of hypovolaemic shock. PHA1 can be acquired, typically in association with pyelonephritis and/or urinary obstruction. There are two inherited forms and the autosomal recessive variant (due to defects in the genes encoding the epithelial sodium channel ENaC) can also be associated with extrarenal manifestations, such as elevated sweat sodium and a skin rash called miliaria and cystic fibrosis-like pulmonary problems.
  4. This disorder can be associated with sensorineural deafness. The biochemical pattern of hypokalaemic, hyperchloraemic acidosis is a characteristic for renal tubular acidosis, and the elevated urine pH indicates dRTA. An elevated plasma sodium concentration reflects water loss and does not exclude a salt-wasting disorder. The frequent wet nappies, the low urinary specific gravity and the hypernatraemia suggest a urinary concentrating defect, which is commonly associated with dRTA. The forms of dRTA associated with genes encoding proton pump subunits or the transcription factor FOXI1 are all associated with sensorineural deafness. More than 90% of patients with dRTA have nephrocalcinosis, reflecting the acidosis-mediated high urinary calcium excretion [31].
  5. The elevated blood pressure suggests a salt-retaining disorder. This girl has a diagnosis of apparent mineralocorticoid excess, due to excessive stimulation of the mineralocorticoid receptor by cortisol with consequent salt retention and hypervolaemia. While the history of prematurity and polyuria and the biochemical pattern of hypokalaemic, hypochloraemic alkalosis and the hypercalciuria are also consistent with a diagnosis of Bartter syndrome, the high blood pressure is not. A normal FENa neither excludes a salt-wasting, nor a salt-retaining disorder, as in a steady-state sodium excretion must balance sodium intake. In this case, the salt retention leads to volume expansion until a new steady state is reached, as the volume expansion leads to decreased proximal sodium reabsorption (hence the hypercalciuria).

## References

[1] Bernard C (1878) Leçons sur les phénomènes de la vie communs aux animaux et aux vegetaux

[2] Cannon WB (1928). Organization for physiological homeostasis. Physiol Rev.

[3] Smith H (1959) From fish to philosopher; the story of our internal environment. Summit, N. J.

[4] Bockenhauer D, Medlar AJ, Ashton E, Kleta R, Lench N (2012). Genetic testing in renal disease. Pediatr Nephrol.

[5] Lifton RP, Gharavi AG, Geller DS (2001). Molecular mechanisms of human hypertension. Cell.

[6] Bockenhauer D, Aitkenhead H (2011). The kidney speaks: Interpreting urinary electrolytes. Arch Dis Child Educ Pract Ed.

[7] Rees L, Bockenhauer D, Webb NJA, Punaro MG (2019) Paediatric nephrology. Oxford University Press

[8] Kleta R, Bockenhauer D (2006). Bartter syndromes and other salt-losing tubulopathies. Nephron Physiol.

[9] Walsh PR, Tse Y, Ashton E, Iancu D, Jenkins L, Bienias M, Kleta R, Van't Hoff W, Bockenhauer D (2018). Clinical and diagnostic features of Bartter and Gitelman syndromes. Clin Kidney J.

[10] Viering D, de Baaij JHF, Walsh SB, Kleta R, Bockenhauer D (2017). Genetic causes of hypomagnesemia, a clinical overview. Pediatr Nephrol.

[11] Haisch L, Konrad M (2012). Impaired paracellular ion transport in the loop of Henle causes familial hypomagnesemia with hypercalciuria and nephrocalcinosis. Ann N Y Acad Sci.

[12] de Baaij JH, Hoenderop JG, Bindels RJ (2015). Magnesium in man: implications for health and disease. Physiol Rev.

[13] Downie ML, Lopez Garcia SC, Kleta R, Bockenhauer D (2020). Inherited Tubulopathies of the kidney: insights from genetics. Clin J Am Soc Nephrol.

[14] Adalat S, Hayes WN, Bryant WA, Booth J, Woolf AS, Kleta R, Subtil S, Clissold R, Colclough K, Ellard S, Bockenhauer D (2019). HNF1B mutations are associated with a gitelman-like tubulopathy that develops during childhood. Kidney Int Rep.

[15] Scholl UI, Dave HB, Lu M, Farhi A, Nelson-Williams C, Listman JA, Lifton RP (2012). SeSAME/EAST syndrome--phenotypic variability and delayed activity of the distal convoluted tubule. Pediatr Nephrol.

[16] Lifton RP, Wilson FH, Choate KA, Geller DS (2002). Salt and blood pressure: new insight from human genetic studies. Cold Spring Harb Symp Quant Biol.

[17] Bockenhauer D, Bichet DG (2013). Inherited secondary nephrogenic diabetes insipidus: concentrating on humans. Am J Physiol Renal Physiol.

[18] Bockenhauer D, van't Hoff W, Dattani M, Lehnhardt A, Subtirelu M, Hildebrandt F, Bichet DG (2010). Secondary nephrogenic diabetes insipidus as a complication of inherited renal diseases. Nephron Physiol.

[19] Nandagopal R, Vaidyanathan P, Kaplowitz P (2009). Transient pseudohypoaldosteronism due to urinary tract infection in infancy: a report of 4 cases. Int J Pediatr Endocrinol.

[20] Kleta R, Bockenhauer D (2018). Salt-losing tubulopathies in children: what’s new, what’s controversial?. J Am Soc Nephrol.

[21] O'Shaughnessy KM (2015). Gordon Syndrome: a continuing story. Pediatr Nephrol.

[22] Arroyo JP, Ronzaud C, Lagnaz D, Staub O, Gamba G (2011). Aldosterone paradox: differential regulation of ion transport in distal nephron. Physiology (Bethesda).

[23] Terker AS, Zhang C, Erspamer KJ, Gamba G, Yang CL, Ellison DH (2016). Unique chloride-sensing properties of WNK4 permit the distal nephron to modulate potassium homeostasis. Kidney Int.

[24] Klootwijk ED, Reichold M, Unwin RJ, Kleta R, Warth R, Bockenhauer D (2015). Renal Fanconi syndrome: taking a proximal look at the nephron. Nephrol Dial Transplant.

[25] Kari JA, El Desoky SM, Singh AK, Gari MA, Kleta R, Bockenhauer D (2014). The case | renal tubular acidosis and eye findings. Kidney Int.

[26] Igarashi T, Inatomi J, Sekine T, Cha SH, Kanai Y, Kunimi M, Tsukamoto K, Satoh H, Shimadzu M, Tozawa F, Mori T, Shiobara M, Seki G, Endou H (1999). Mutations in SLC4A4 cause permanent isolated proximal renal tubular acidosis with ocular abnormalities. Nat Genet.

[27] Rodriguez Soriano J (2002). Renal tubular acidosis: the clinical entity. J Am Soc Nephrol.

[28] Gale DP, Mallett A, Patel C, Sneddon TP, Rehm HL, Sampson MG, Bockenhauer D (2020). Diagnoses of uncertain significance: kidney genetics in the 21st century. Nat Rev Nephrol.

[29] Ashton EJ, Legrand A, Benoit V, Roncelin I, Venisse A, Zennaro MC, Jeunemaitre X, Iancu D, Van't Hoff WG, Walsh SB, Godefroid N, Rotthier A, Del Favero J, Devuyst O, Schaefer F, Jenkins LA, Kleta R, Dahan K, Vargas-Poussou R, Bockenhauer D (2018). Simultaneous sequencing of 37 genes identified causative mutations in the majority of children with renal tubulopathies. Kidney Int.

[30] Ashton E, Bockenhauer D (2020). Diagnosis of uncertain significance: can next-generation sequencing replace the clinician?. Kidney Int.

[31] Lopez-Garcia SC, Emma F, Walsh SB, Fila M, Hooman N, Zaniew M, Bertholet-Thomas A, Colussi G, Burgmaier K, Levtchenko E, Sharma J, Singhal J, Soliman NA, Ariceta G, Basu B, Murer L, Tasic V, Tsygin A, Decramer S, Gil-Pena H, Koster-Kamphuis L, La Scola C, Gellermann J, Konrad M, Lilien M, Francisco T, Tramma D, Trnka P, Yuksel S, Caruso MR, Chromek M, Ekinci Z, Gambaro G, Kari JA, Konig J, Taroni F, Thumfart J, Trepiccione F, Winding L, Wuhl E, Agbas A, Belkevich A, Vargas-Poussou R, Blanchard A, Conti G, Boyer O, Dursun I, Pinarbasi AS, Melek E, Miglinas M, Novo R, Mallett A, Milosevic D, Szczepanska M, Wente S, Cheong HI, Sinha R, Gucev Z, Dufek S, Iancu D, Kleta R, Schaefer F, Bockenhauer D, European d RTA Consortium (2019). Treatment and long-term outcome in primary distal renal tubular acidosis. Nephrol Dial Transplant.

